# Constructing ‘Molecular Bridges’ Between Aramid Pulp and Rubber for Significantly Enhanced Reinforcement

**DOI:** 10.3390/polym18212685

**Published:** 2026-10-29

**Authors:** Fei Zhao, Jiawei Ding, Ruiheng Jin, Shihao Wang, Feifan Duan, Hongyue Wu, Guohua Sun, Minglei Pang, Zhiyong Sun, Weidong Ma

**Affiliations:** Shandong Non-Metallic Materials Research Institute, Jinan 250031, China

**Keywords:** aramid pulp, surface grafting, thiol functionalization, interfacial co-crosslinking

## Abstract

Aramid pulp (AP), a short fiber reinforcement with excellent mechanical properties, is highly promising for weight reduction and reinforcement in rubber transmission belts, tires, and sealing products. However, its smooth and chemically inert surface leads to weak interfacial adhesion with the rubber matrix, severely limiting its reinforcing efficiency. In this study, a two-step surface grafting strategy combining alkaline treatment, isocyanate activation, and thiol functionalization was proposed, through which thiol groups capable of co-vulcanization with nitrile butadiene rubber (NBR) were successfully introduced onto the aramid pulp surface. The effects of AP at different treatment stages on the mechanical properties and dynamic service behavior of NBR composites were systematically evaluated. The results demonstrated that, compared with the unmodified AP, the NBR composite reinforced with the two-step modified AP exhibited a significantly higher tensile modulus, improved abrasion resistance, a greatly prolonged tensile fatigue life at 50% strain, and reduced compression heat build-up. This work demonstrates that the interfacial co-crosslinking strategy is effective in enhancing the performance of aramid pulp-reinforced rubber and provides experimental evidence and new insights for the interfacial design of high-performance aramid fiber/rubber composites.

## 1. Introduction

Rubber-based composites are extensively utilized in aerospace, automotive manufacturing, and high-end sports equipment due to their excellent flexibility, damping properties, and fatigue resistance [1,2]. However, the strength and modulus of neat rubber matrices often fail to meet the stringent requirements of modern engineering structures for lightweight yet high-strength materials; thus, the addition of reinforcing fillers is indispensable in rubber processing [3,4,5]. Among various reinforcing fillers, AP is regarded as an ideal high-performance alternative to traditional carbon black and silica, owing to its unique fibrillated three-dimensional structure, extremely high tensile strength (~3 GPa) and modulus (~130 GPa), and a density comparable to that of the rubber matrix. Its reinforcing potential has garnered significant attention, particularly in the fields of green tires and specialty seals [6,7].

Although AP possesses exceptional intrinsic mechanical properties, poor interfacial compatibility with the rubber matrix has long been a bottleneck limiting composite performance [8,9,10]. AP has a surface composed of highly crystalline aromatic polyamide chains with high chemical inertness and few active functional groups [11,12,13]. Consequently, interactions with non-polar or weakly polar rubbers (such as natural rubber (NR) and styrene–butadiene rubber (SBR)) are limited to weak van der Waals forces [14,15,16]. Under dynamic loads or hygrothermal environments, debonding and slippage readily occur at the interface, leading to inefficient stress transfer and severely restricting the realization of AP’s reinforcing potential [17,18,19]. Therefore, chemically functionalizing the AP surface to construct a robust interfacial phase is key to achieving breakthroughs in the performance of rubber composites [20,21,22].

Current mainstream strategies for AP surface modification include plasma treatment, high-energy irradiation grafting, and silane coupling agent coating [23,24,25]. While plasma treatment can introduce hydroxyl or carboxyl groups, the modified layer is extremely thin and prone to rearrangement failure during storage. Silane coupling agent methods are often limited by the lack of reactive silanol groups on the AP surface, resulting in incomplete hydrolysis and condensation of the coupling agent and insufficient interfacial adhesion [26]. In recent years, isocyanate-based chemical grafting has attracted attention due to its high efficiency and strong bond energy; however, precisely introducing reactive groups capable of chemically coupling with the rubber vulcanization network onto the fiber surface without compromising the bulk strength of AP remains an unresolved challenge [27,28].

Inspired by the concept of “molecular bridge” construction, this study proposes a mild and controllable stepwise chemical grafting strategy aimed at building a reactive interfacial layer on the AP surface compatible with rubber vulcanization [29,30]. First, a limited hydrolysis reaction under alkaline conditions was employed to introduce highly active aromatic amino groups onto the AP surface [31,32]. Subsequently, toluene diisocyanate (TDI) was used as a bifunctional chain extender; its highly reactive isocyanate groups (-NCO) underwent nucleophilic addition with the surface amino groups to construct isocyanate-terminated active intermediates [33]. Finally, pentaerythritol tetrakis(3-mercaptopropionate) (PETMP), a multifunctional monomer containing thiol groups, was selected to react with the terminal isocyanate groups of the intermediate, successfully grafting thiol groups with high reactivity onto the AP surface [34].

Through the precise regulation of this “hydrolysis–amidation–TDI chain extension–thiol termination” process, the surface of the modified AP is endowed with dense reactive “anchor hooks”. During the subsequent mixing and vulcanization with latex, these surface thiol groups may participate in the rubber cross-linking network, potentially forming an interpenetrating network structure between the fiber and rubber. This study not only provides a novel and efficient method for the chemical surface modification of aramid but also offers theoretical basis and technical support for solving the interfacial debonding problem between high-performance fibers and rubber matrices, promising significant improvements in the wear resistance and dynamic mechanical properties of rubber composites.

## 2. Experimental Section

### 2.1. Materials

AP (Taparan 1820) was supplied by Yantai Tayho Advanced Materials Co., Ltd, Yantai, China. TDI and PETMP were purchased from Macklin Biochemical Co., Ltd, Shanghai, China. NBR latex with a solid content of 44 wt% was provided by Lanzhou Petrochemical Company, PetroChina, Lanzhou, China. All other reagents and raw materials used in this work were of analytical grade and obtained from standard commercial suppliers.

### 2.2. Sample Preparation

#### 2.2.1. Surface Cleaning

10 g of AP was placed in a round-bottom flask, followed by the addition of 300 mL of acetone. The mixture was ultrasonicated in a water bath for 30 min. The pulp was then separated via vacuum filtration and dried at 60 °C. The sample is denoted as AP.

#### 2.2.2. Alkaline Hydrolysis

The cleaned AP was dispersed in a mixed solvent system (300 mL, ethanol:water = 3:1) containing 33.7 g of potassium hydroxide (KOH). The suspension was heated to 80 °C with constant stirring and maintained for 1 h. After cooling, the pulp was filtered, thoroughly washed with deionized water until the filtrate reached neutral pH, and subsequently dried at 60 °C. The sample was denoted as APK (AP after KOH hydrolysis).

#### 2.2.3. TDI Grafting

The hydrolyzed pulp was placed in a flask with 300 mL of acetone, and 4 mL of TDI was introduced. The reaction system was heated to reflux with continuous stirring for 2 h. Upon cooling, the solid was isolated by filtration and washed three times with fresh acetone. The sample was denoted as APKT (APK after TDI grafting).

#### 2.2.4. Thiol Functionalization

The TDI-grafted pulp was transferred to a flask containing 300 mL of acetone and 2 mL of PETMP. The mixture was heated to reflux with constant stirring for 2 h. After cooling, the product was filtered and washed three times with anhydrous ethanol. The sample was denoted as APKTS (APKT after thiol functionalization with PETMP).

#### 2.2.5. Latex Co-Coagulation and Masterbatch Preparation

The functionalized pulp was dispersed in 250 mL of an ethanol/water mixture (1:1 *v*/*v*). This dispersion was blended uniformly with 250 mL of a diluted NBR latex (solid content: 40 g). A 10% calcium chloride solution was added dropwise under stirring to induce demulsification and flocculation. The resulting solid was passed through a two-roll mill to remove excess moisture and subsequently dried at 60 °C to obtain the modified AP/NBR masterbatch. AP, APK, APKT, and APKTS were all prepared into masterbatches using the same procedure.

### 2.3. Measurements

A universal testing machine (HDW-2H, Jinan Liling Testing Machine Co., Ltd., Jinan, China) was used; it can precisely record the strain of rubber. Tensile properties were measured according to ISO 37-2005 [35], and tear strength was calculated according to ISO 34-1-2010 (right angle, [36]). All the values were calculated by taking the average of five samples. Fourier transform infrared (FTIR) spectrometer: ALPHA II, Bruker, Billerica, USA. Scanning electron microscope (SEM): Gemini SEM 300, Carl Zeiss, Oberkochen, Germany. X-ray photoelectron spectrometer (XPS): ESCALAB QXi, Thermo Scientific, Waltham, USA. A Tougaard-type background was applied for all high-resolution spectra (C 1s, N 1s, O 1s, and S 2p) during the least-squares fitting procedure. A combined Gaussian–Lorentzian line shape function was used for all component peaks. The spectra were charge-corrected using the C 1s peak at 284.8 eV as a reference. During the fitting process, the constraints, FWHM values (typically constrained within 0.8–1.6 eV for all deconvoluted peaks), and other fitting parameters were kept consistent across all samples. The curing characteristics of the rubber compound were measured using a D-RPA 3000 (MonTech, Buchen, Germany) at 160 °C according to ISO 6502-1:2018 [37]. The tan δ, torque, and other parameters were automatically calculated by the software. The Akron abrasion test was carried out in accordance with GB/T 1689-2014 [38], and each sample was tested three times. The tension fatigue life test and specimen preparation were carried out in accordance with GB/T 1688-2008 [39]. The test was performed using a Tensile & Shearing Fatigue Machine manufactured by Beijing RADE Instrument Co., Ltd, Beijing, China. The specimen thickness was 2 mm, the test frequency was 4 Hz, and the test temperature was room temperature. The test was continued until the specimen completely fractured. The compression heat build-up of the specimens was tested in accordance with GB/T 1687.4-2021 [40], with a static force of 350 N, a dynamic force of 250 N, and a vibration frequency of 20 Hz. The DMA tests of the specimens were performed using a GABO EPLEXOR high-force dynamic mechanical analyzer manufactured by NETZSCH (Selb, Germany), with a static strain of 10%, a dynamic strain of 1%, and a frequency of 10 Hz.

The NBR rubber formulation used is shown in Table 1 below.

All tested samples were prepared using the same procedure. The mixing sequence was as follows: NBR was first mixed in an internal mixer for 1 min (35 rpm). Then zinc oxide, stearic acid, antioxidant, and AP masterbatch were added, and mixing was continued for another 1 min. Subsequently, carbon black and plasticizer were added, and mixing was continued for 3 min, during which the chamber temperature was maintained below 100 °C. The compound was discharged and sheeted on an open mill, then cooled for 12 h. Finally, sulfur and accelerator were incorporated on the open mill. After sulfur addition, the compound was allowed to mature for 24 h and then compression-molded in a platen vulcanizing press at a curing temperature of 160 °C and a pressure of 10 MPa.

## 3. Results and Discussion

The construction of molecular bridges between AP and the rubber phase is achieved through a two-step grafting process, as illustrated in Figure 1. First, the AP is treated with KOH solution to hydrolyze the surface amide groups, generating amine groups containing active hydrogen atoms. Subsequently, TDI is introduced to undergo an addition reaction with these amine groups, forming urea groups and thereby grafting unreacted isocyanate groups onto the AP surface. The isocyanate groups are then reacted with the thiol groups of PETMP, further grafting thiol groups onto the AP surface that are capable of undergoing click chemistry with the double bonds in the rubber. Finally, the modified AP is mixed with NBR latex to produce a masterbatch, which is then compounded with rubber and co-vulcanized, thereby potentially forming molecular bridges between the AP and the rubber phase.

To characterize the effect of the two-step grafting method on AP, the products obtained at different treatment stages were analyzed by FTIR, and the results are shown in Figure 2. As shown by the curve for AP in the figure, the broad absorption peak of AP at 3320 cm^−1^ is attributed to the N–H stretching vibration of amide bonds [19]; the strong absorption peak at 1640 cm^−1^ is assigned to the stretching vibration of the amide carbonyl (C=O) [9]; and the absorption peaks at 1540 cm^−1^ and 1510 cm^−1^ are attributed to the coupling of N–H bending vibration and C–N stretching vibration. In addition, the characteristic absorption peaks near 1600 cm^−1^ and 1500 cm^−1^ confirm the presence of aromatic ring structures. Comparing AP with APK, it can be found that after hydrolysis treatment with KOH solution, no significant changes were observed in the FTIR spectrum, indicating that the KOH hydrolysis method did not cause obvious damage to AP, and the molecular structure of AP remained intact. Compared with APK, APKT did not exhibit the characteristic absorption peak of the isocyanate group, possibly because the isocyanate group reacted with water during storage to form amino groups. The transmission-mode FTIR spectra mainly reflect the bulk composition of the aramid pulp. Because the surface modification layer is extremely thin compared with the fiber bulk, its contribution to the transmission FTIR spectra is negligible, which explains the close similarity of the spectra of AP, APK, APKT, and APKTS. By comparing APKTS with APKT, a characteristic absorption peak of the carbonyl group (C=O) can be observed near 1725 cm^−1^. This carbonyl group may originate from PETMP, indicating that thiol-rich PETMP macromolecules may have been grafted onto the AP surface [41,42].

To investigate the morphological changes in AP during the stepwise grafting modification process, SEM was employed to observe the microstructure of AP, and the results are shown in Figure 3. The pristine AP (Figure 3a) exhibited a typical fibrillated morphology, where numerous fibrils were generated from the aramid fibers during the beating process, and the original aramid fibers displayed many cracks and defects due to mechanical compression. After hydrolysis treatment with KOH solution (APK, Figure 3b), the microstructure of AP remained largely unchanged. For the samples after grafting TDI and PETMP (APKTS, Figure 3c,d), no obvious changes in microstructure were observed, which can be attributed to the fact that the grafting of TDI and PETMP occurred at the molecular layer on the fiber surface and thus did not exert a noticeable influence on the microscopic morphology.

To further elucidate the evolution of surface chemical states on AP during each modification step, XPS characterization was conducted on all samples, and the results are presented in Figure 4. As shown in Figure 4a, the survey spectra of AP, APK, and APKT exhibit no discernible differences, which is expected given that neither KOH hydrolysis nor TDI grafting introduces any heteroatoms. In contrast, a distinct S 2p signal was detected in the APKTS sample, confirming the successful grafting of PETMP and the consequent introduction of thiol groups onto the AP surface.

The high-resolution C 1s spectra of all samples were deconvoluted into four peaks, corresponding to carbon in phenyl rings (C Ph, 284.6 eV), carbon linked to amide groups (C–N, 284.8 eV), carbon bonded to oxygen in amide groups (C=O, 287.6 eV), and carbon in carboxylic acid groups (O–C=O, 289.9 eV) [43]. The integrated areas and their ratios for each sample are summarized in Table 2. Comparison of the O–C=O/C Ph and C=O/C–Ph ratios reveals that after KOH hydrolysis, the proportion of O–C=O increased relative to AP, indicating the generation of additional carboxylic acid groups. Following TDI grafting, the C=O/C–Ph ratio increased markedly, attributable to the introduction of supplementary C=O bonds from TDI. After subsequent PETMP grafting, the O–C=O/C–Ph ratio further increased, arising from the ester groups inherent to PETMP. Moreover, as shown in Figure 4c, the integrated N 1s peak area of APKT is larger than those of both AP and APK, providing additional evidence for the successful grafting of TDI onto the AP surface.

As shown in Figure 4d, the O 1s spectra were fitted into two peaks, assigned to oxygen in amide groups (O=C, 532.5 eV) and oxygen in carboxylic acid groups (O–C, 531.0 eV). The corresponding integrated areas and ratios are listed in Table 3. Notably, the O-C/O=C ratio of APK is higher than that of AP, indicating that KOH hydrolysis promoted the formation of carboxylic acid groups on the AP surface. Similarly, the O-C/O=C ratio in APKTS is also elevated relative to AP, which can be attributed to the introduction of ester groups from PETMP [44]. Finally, as shown in Figure 4e, a well-defined S 2p doublet (S 2p_3_/_2_ at 163.5 eV and S 2p_1_/_2_ at 164.7 eV) was observed exclusively in the APKTS sample, confirming the presence of sulfur in the grafted product and providing clear evidence for the successful functionalization of AP with PETMP.

The surface elemental compositions of AP, APK, APKT, and APKTS were determined by XPS, and the results are expressed in atomic percentage. As shown in Table 4, after KOH hydrolysis and subsequent TDI grafting, the surface sulfur content showed essentially no increase compared with that of pristine aramid pulp. In contrast, after further grafting with PETMP, the sulfur atomic percentage increased markedly, further confirming that PETMP was grafted onto the aramid pulp surface.

The curing characteristics of aramid pulp-reinforced NBR at different treatment steps were evaluated (Table 5). A comparison reveals that the addition of aramid pulp prolonged the scorch time of the compound, while the optimum cure time was slightly reduced. When comparing the aramid pulp samples subjected to different treatments, the effects of the treatment steps on the scorch time and optimum cure time are negligible, and the influences on the minimum torque, maximum torque, and loss factor (tan δ) are also relatively small. Therefore, the two-step grafting method of aramid pulp has a minor effect on the vulcanization of the reinforced rubber.

To investigate the effect of constructing molecular bridges at the AP–rubber interface on the mechanical properties of the reinforced rubber, tensile tests were performed on NBR reinforced with AP at different treatment stages, and the results are presented in Figure 5. It can be observed from Figure 5 that the addition of AP significantly increased the modulus at strains of 0–100%. However, at strains beyond 200%, the stresses of the NBR/AP, NBR/APK, and NBR/APKT samples were even lower than that of neat NBR. This may be attributed to debonding between the aramid pulp fibers and the rubber matrix. In contrast, the stress–strain curve of the APKTS lay entirely above that of NBR, indicating that the two-step grafting approach promoted stronger interfacial adhesion between the AP fibers and the rubber matrix.

The mechanical property data and standard deviations of all samples are presented in Table 6. Comparison shows that after the addition of AP, the 100% modulus of the samples is significantly enhanced. Moreover, no significant difference in the 100% modulus is observed after KOH treatment (APK) and the first grafting step of AP (APKT), whereas after PETMP grafting, the 100% modulus of the APKTS sample is markedly improved. Further comparison reveals that only the APKTS sample exhibits higher at 200% and 300% modulus and higher tensile strength than the neat NBR sample, while the other samples that did not undergo the two-step grafting show decreases in these properties. For APKTS, the stronger interfacial adhesion may delay debonding and facilitate stress transfer, thereby causing its stress–strain curve to lie above that of neat NBR. This further suggests that the thiol groups on AP may participate in the formation of crosslinks with the rubber phase.

The tear strength of NBR reinforced with AP under different treatment processes was evaluated, and the results are presented in Table 7. After the addition of aramid pulp, the tear strength of all samples exhibited a slight improvement, although the degree of enhancement was limited. Moreover, whether the AP was modified had no significant effect on the improvement in tear strength. This phenomenon may be attributed to the fact that during the tear test, the specimen first undergoes considerable deformation, and crack initiation and subsequent crack propagation occur very rapidly, in a manner similar to the tensile fracture process; consequently, the results are more comparable with those of the tensile test.

To further investigate the effect of the two-step grafting method on the aramid–rubber interface, the crosslink densities of NBR samples reinforced with AP at different treatment stages were measured. The crosslink densities of the samples determined by low-field nuclear magnetic resonance (LF-NMR) are summarized in Table 8. Comparison reveals that the crosslink density of the vulcanized rubber increased slightly after the addition of aramid pulp, which may be attributed to the enhancement of the physical crosslink density of the rubber molecules by the added aramid pulp. The NBR/APKTS sample exhibited the highest crosslink density, which may be caused by the formation of chemical bonds between the surface thiol groups and the rubber.

Elongation fatigue tests were conducted on AP-reinforced NBR subjected to different treatment processes under various strains, and the results are shown in Figure 6. It can be observed that, after the addition of AP, the fatigue life at 100% strain decreased significantly compared to that of the neat NBR sample, and the modification of the AP had only a limited effect on the fatigue life. At 75% strain, the tensile fatigue life of the AP-filled samples was slightly lower than that of pure NBR, and notably, the sample containing APKTS exhibited a superior fatigue life to the other samples. At 50% strain, the fatigue life of the samples with AP increased remarkably compared to the neat NBR, with the APKTS sample showing a significantly higher fatigue life than the others. This indicates that stronger interfacial interactions existed between the AP and the rubber phase after the two-step grafting modification, suggesting that the thiol groups on APKTS may participate in the vulcanization network and contribute to the enhanced interfacial adhesion.

The microscopic images of the fracture surfaces of AP, APK, APKT, and APKTS after 50% tensile fatigue are shown in Figure 7. It can be observed that the fracture surfaces contain aramid pulp fibers, and the aramid fibers are relatively uniformly dispersed in the rubber phase. A further comparison reveals that obvious grooves along the fiber direction can be observed on the fiber surfaces of the AP, APK, and APKT samples. This is a unique structure of the fiber core layer, indicating that less rubber is adhered to the aramid fiber surfaces in the AP, APK, and APKT samples. In contrast, a greater amount of adhered rubber can be observed on the APKTS surface, which further indicates that the interfacial interaction between APKTS and the rubber phase is enhanced.

Akron abrasion tests were performed on NBR samples reinforced with AP subjected to different modification treatments, and the results are presented in Table 9. A comparison of the Akron abrasion data reveals that the addition of AP significantly improved the Akron abrasion resistance of NBR. Further comparison indicates that AP treated with KOH and grafted with TDI did not enhance the abrasion resistance of NBR, whereas AP subsequently grafted with PETMP led to a more noticeable improvement. This demonstrates that the AP grafted with PETMP possesses stronger interfacial interactions with the rubber phase.

To further elucidate the mechanism by which different surface treatments affect the abrasion resistance of the reinforced rubber materials, the surface morphologies of the specimens after the Akron abrasion test were observed by SEM, as shown in Figure 8. The worn surface of the NBR sample exhibits slight tearing of the rubber matrix. For the composites containing unmodified AP and the intermediate products (Figure 8b–d), obvious holes left by pulled-out aramid fibers can be observed on the worn surfaces, and the fiber surfaces are relatively smooth. This indicates that the weaker interfacial adhesion is insufficient to withstand the shear stress during the abrasion process, leading to easy debonding and pull-out of the fibers from the rubber matrix, so that the fibers fail to fully exert their load-bearing capacity. In contrast, for the NBR/APKTS composite (Figure 8e,f), the worn fibers are found to remain embedded in the rubber matrix, indicating tighter bonding between the fibers and the rubber matrix. This morphological transition directly confirms that the two-step grafting modification constructs stronger interfacial adhesion between APKTS and the rubber, inhibiting fiber debonding and detachment during the abrasion process, thereby reducing the Akron abrasion loss.

Compression heat build-up tests were conducted on AP-reinforced NBR at different treatment stages, and the results are presented in Table 10. A comparison reveals that the addition of AP significantly reduced the compression heat build-up of NBR. This can be attributed to the increased modulus of the NBR composite resulting from the incorporation of AP, which reduced the deformation amplitude of the rubber matrix. Furthermore, after the two-step grafting, the NBR/APKTS sample exhibited even lower heat build-up. This finding is consistent with the previous stress–strain curve results, indicating that stronger interfacial interactions were formed between the AP and the rubber phase after the two-step grafting.

To further investigate the effect of two-step graft-modified AP on the dynamic mechanical properties of the reinforced NBR, DMA temperature sweep tests were conducted on all samples from room temperature to 100 °C, and the resulting variation curves of the loss factor (tan δ) with temperature are shown in Figure 9. As observed in the figure, the tan δ of all samples exhibits a gradually decreasing trend with increasing temperature. This is a typical characteristic of rubber materials, attributed to the intensified molecular chain segment mobility and the consequent reduction in hysteresis loss at elevated temperatures. At the same testing temperature, a comparison of the curves reveals that the neat NBR sample exhibits the highest tan δ, whereas the NBR/APKTS sample maintains the lowest, with the other modified samples lying in between. This dynamic mechanical result is consistent with the compression heat build-up results in Table 9. This difference can be attributed to the stronger interfacial interactions constructed between APKTS and the rubber matrix via the two-step grafting modification, which suppresses the sliding and friction of rubber molecular chains on the filler surface, thereby reducing the energy loss during dynamic deformation.

## 4. Conclusions

In this work, a surface grafting strategy combining alkaline treatment, isocyanate activation, and thiol functionalization was employed to successfully introduce thiol groups capable of co-vulcanization with the rubber matrix onto the AP surface. Compared with the unmodified AP and the samples treated only with KOH and TDI, the NBR composite reinforced with APKTS exhibited significantly improved tensile modulus and enhanced abrasion resistance; the elongation fatigue life at 50% strain was greatly extended, while the compression heat build-up was further reduced. These performance improvements are primarily attributed to the participation of the thiol groups introduced by PETMP grafting in the rubber vulcanization process, which likely form covalent crosslinks at the AP–NBR interface. The proposed molecular-bridge mechanism is consistent with the chemical, mechanical, fatigue, abrasion, and heat build-up responses observed in this work. This effectively enhanced interfacial stress transfer and suppressed the frictional slippage of molecular chains at the interface, thereby endowing the composite with superior overall performance under both static and dynamic loading. This work demonstrates that the construction of co-crosslinkable interfacial molecular bridges can substantially improve the reinforcing effect of AP on NBR, providing new insights and experimental evidence for the interfacial design of high-performance aramid fiber/rubber composites.

## Figures and Tables

**Figure 1:**
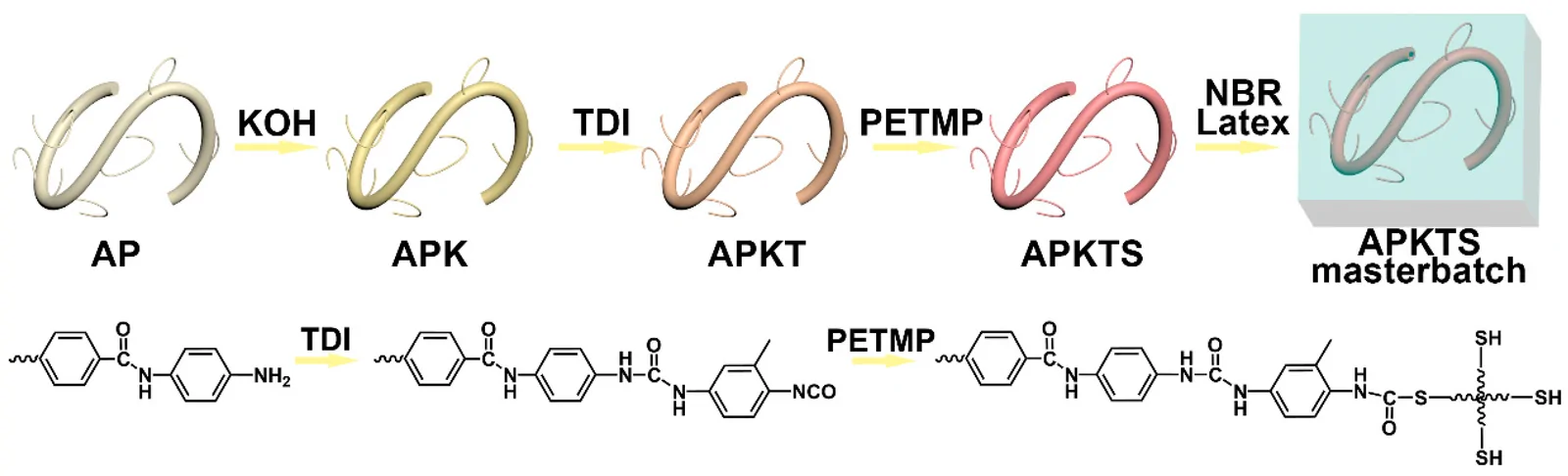
Schematic diagram of the two-step grafting process and mechanism for AP.

**Figure 2:**
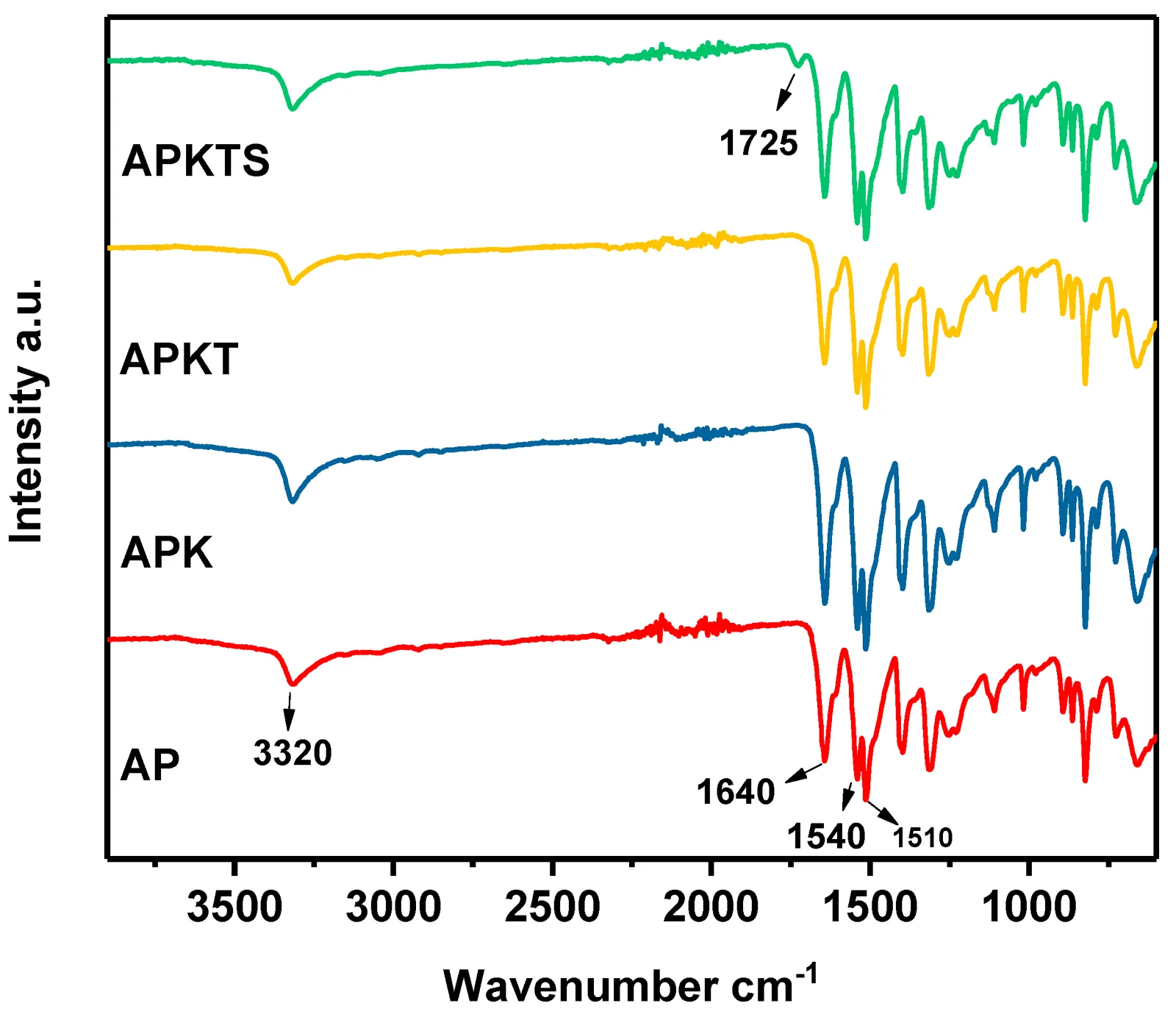
FTIR spectra of AP, APK, APKT, and APKTS.

**Figure 3:**
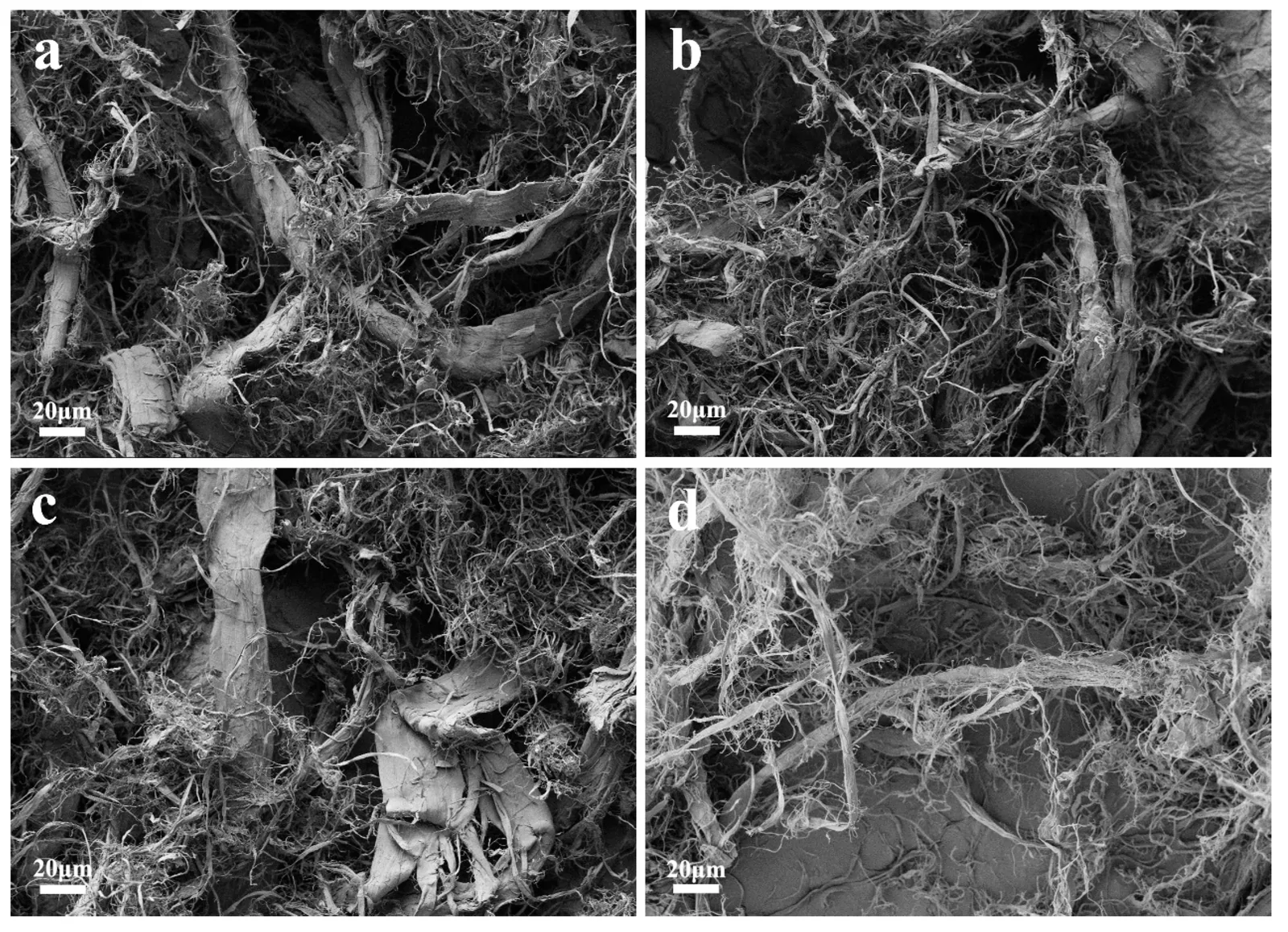
SEM images of (**a**) AP, (**b**) APK, (**c**) APKT, and (**d**) APKTS.

**Figure 4:**
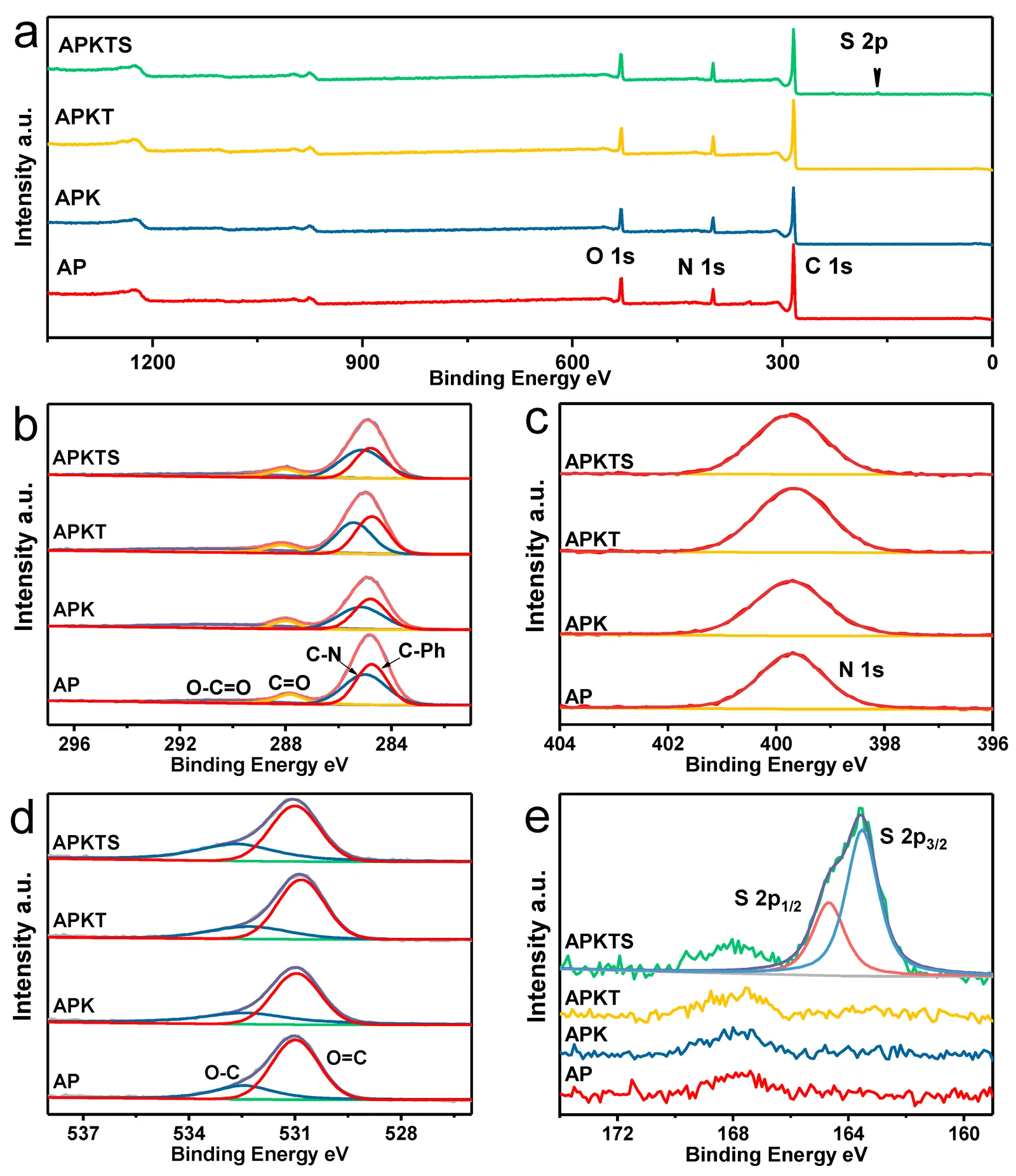
(**a**) XPS survey spectra of AP, APK, APKT, and APKTS samples; (**b**–**e**) high-resolution XPS spectra of C 1s, N 1s, O 1s, and S 2p regions for each sample, respectively.

**Figure 5:**
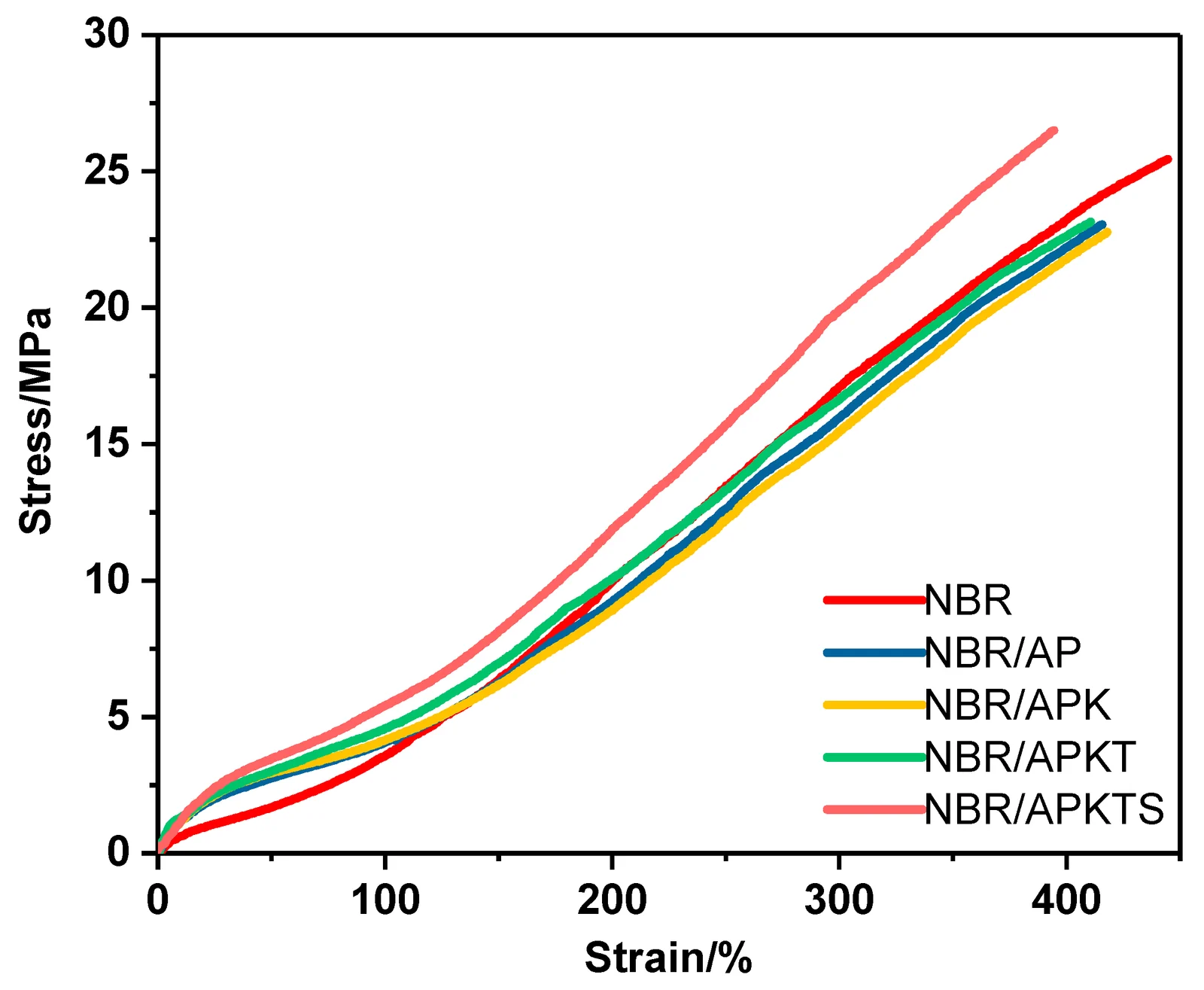
Stress–strain curves of NBR reinforced with AP under different treatment processes.

**Figure 6:**
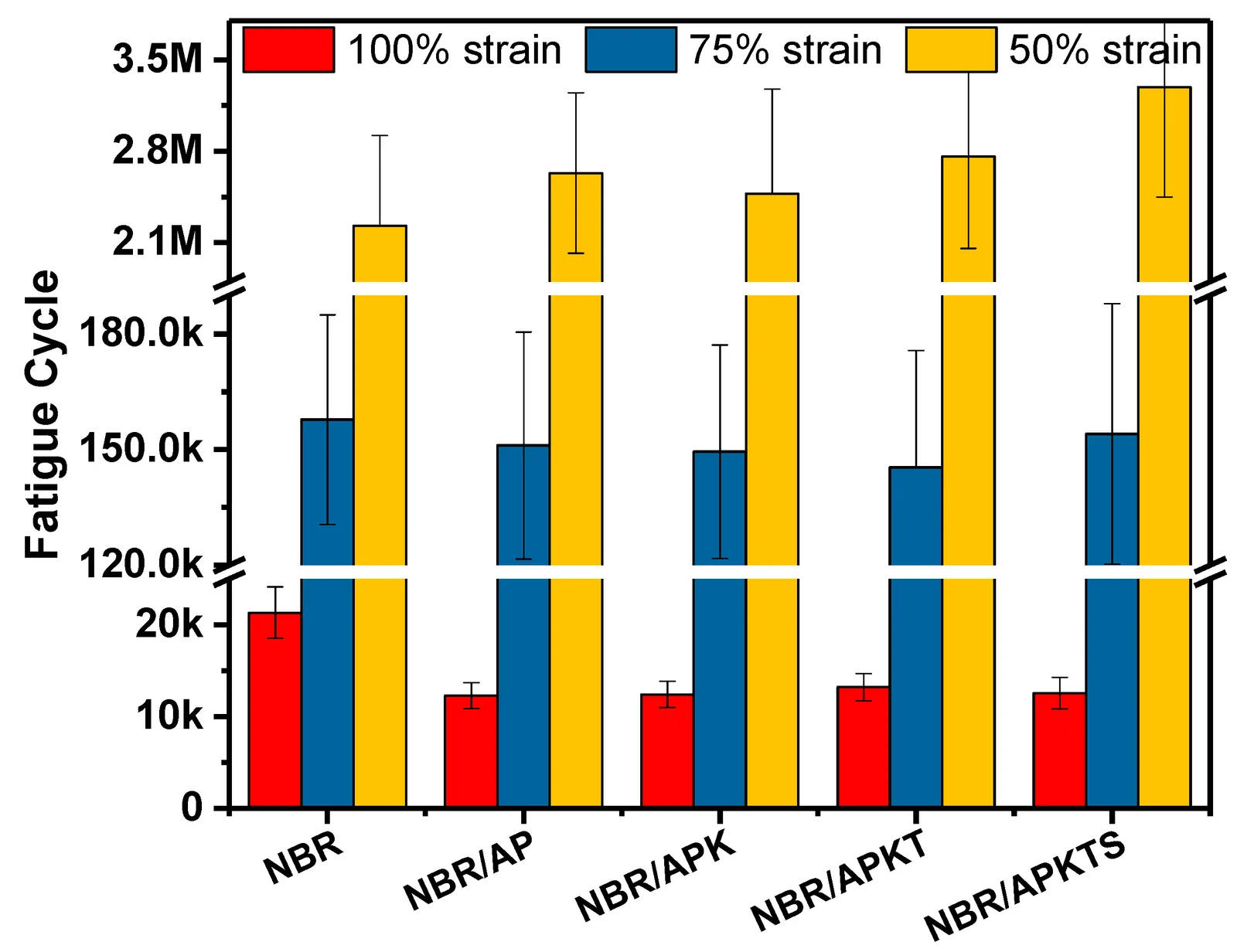
Fatigue life at different elongation strains of NBR reinforced with AP under different treatment processes.

**Figure 7:**
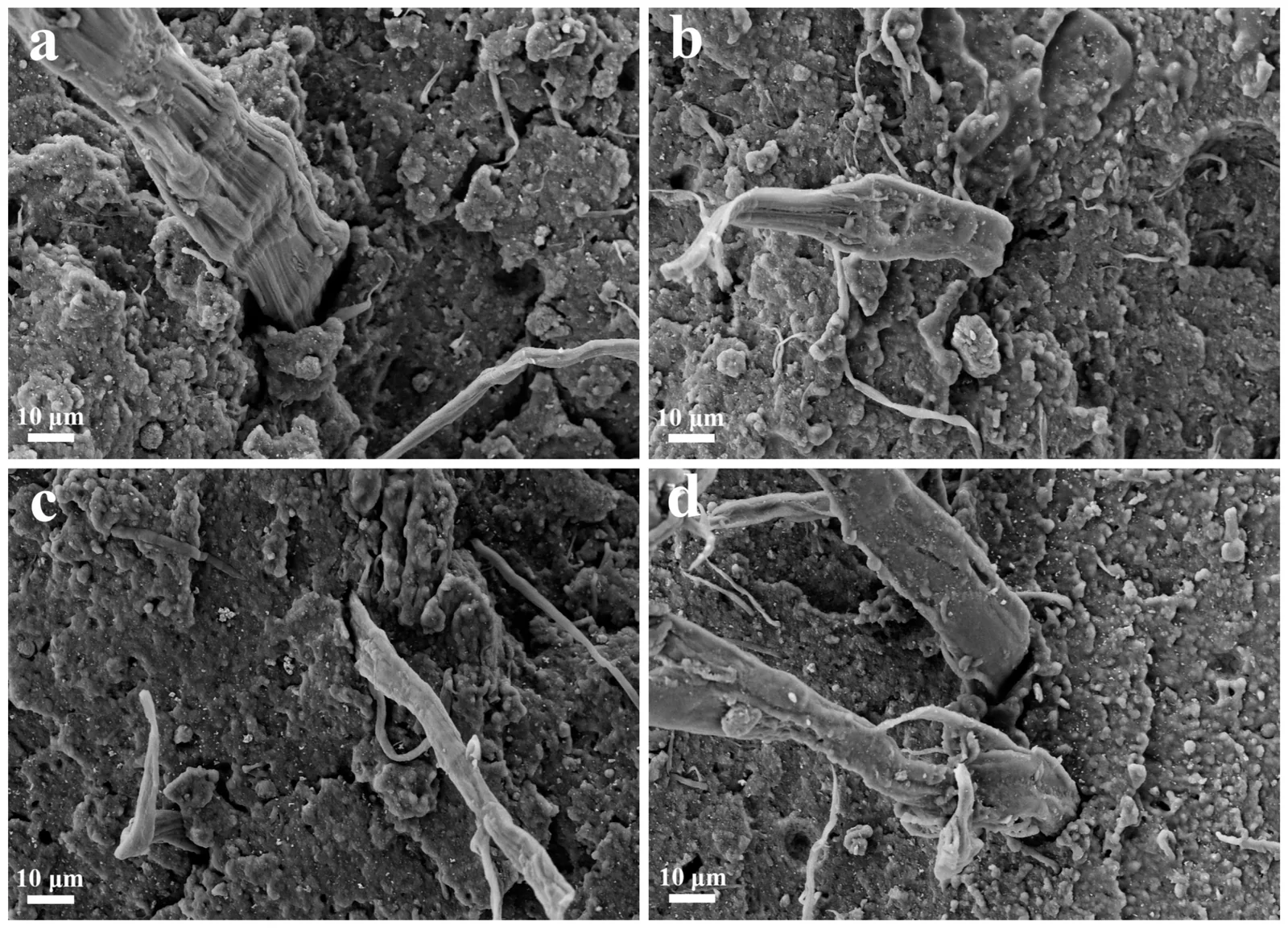
SEM images of the 50% tensile fatigue fracture surfaces: (**a**) AP, (**b**) APK, (**c**) APKT, and (**d**) APKTS.

**Figure 8:**
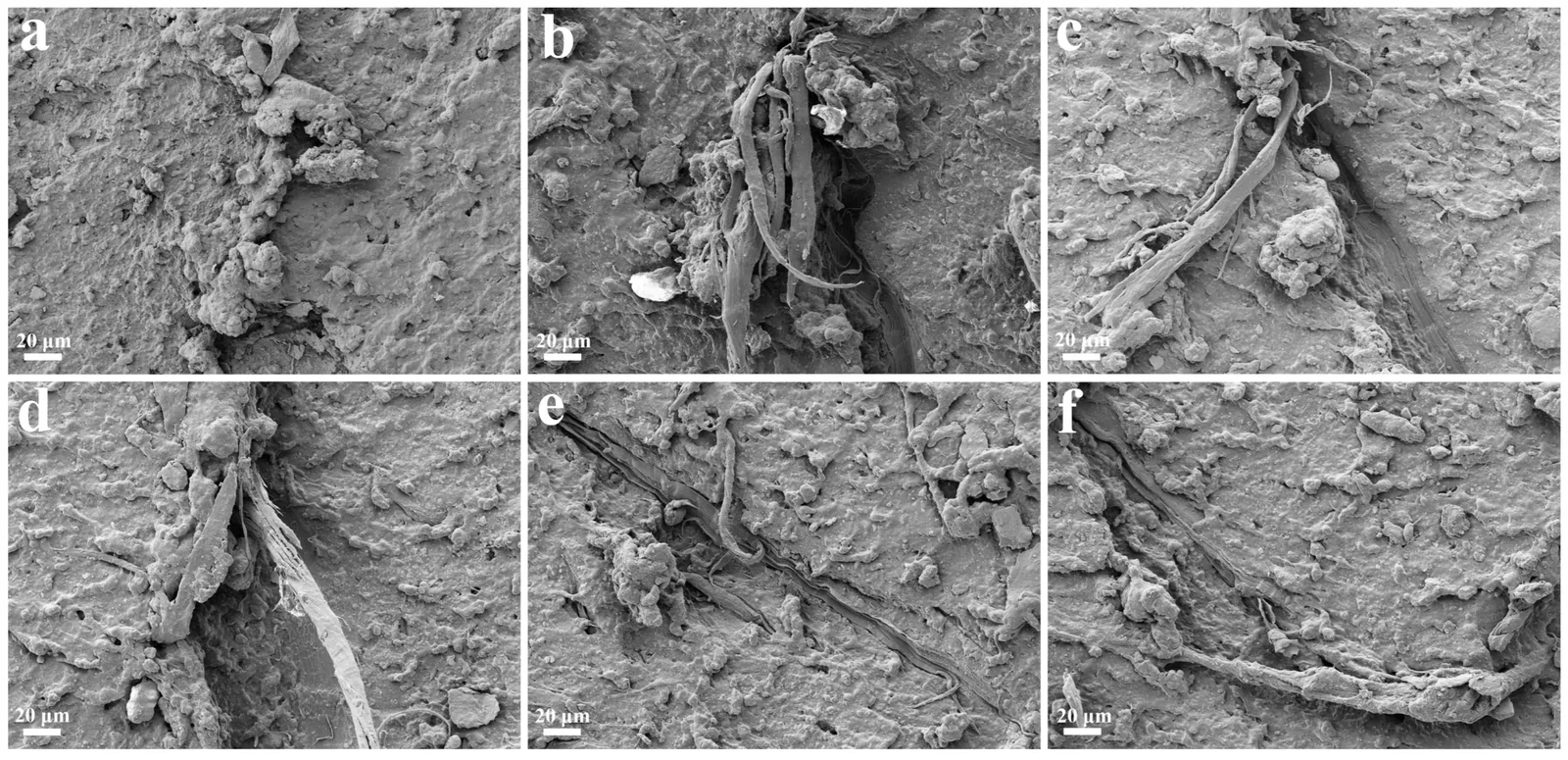
SEM images of the worn surfaces after the Akron abrasion test: (**a**) NBR, (**b**) NBR/AP, (**c**) NBR/APK, (**d**) NBR/APKT, and (**e**,**f**) NBR/APKTS.

**Figure 9:**
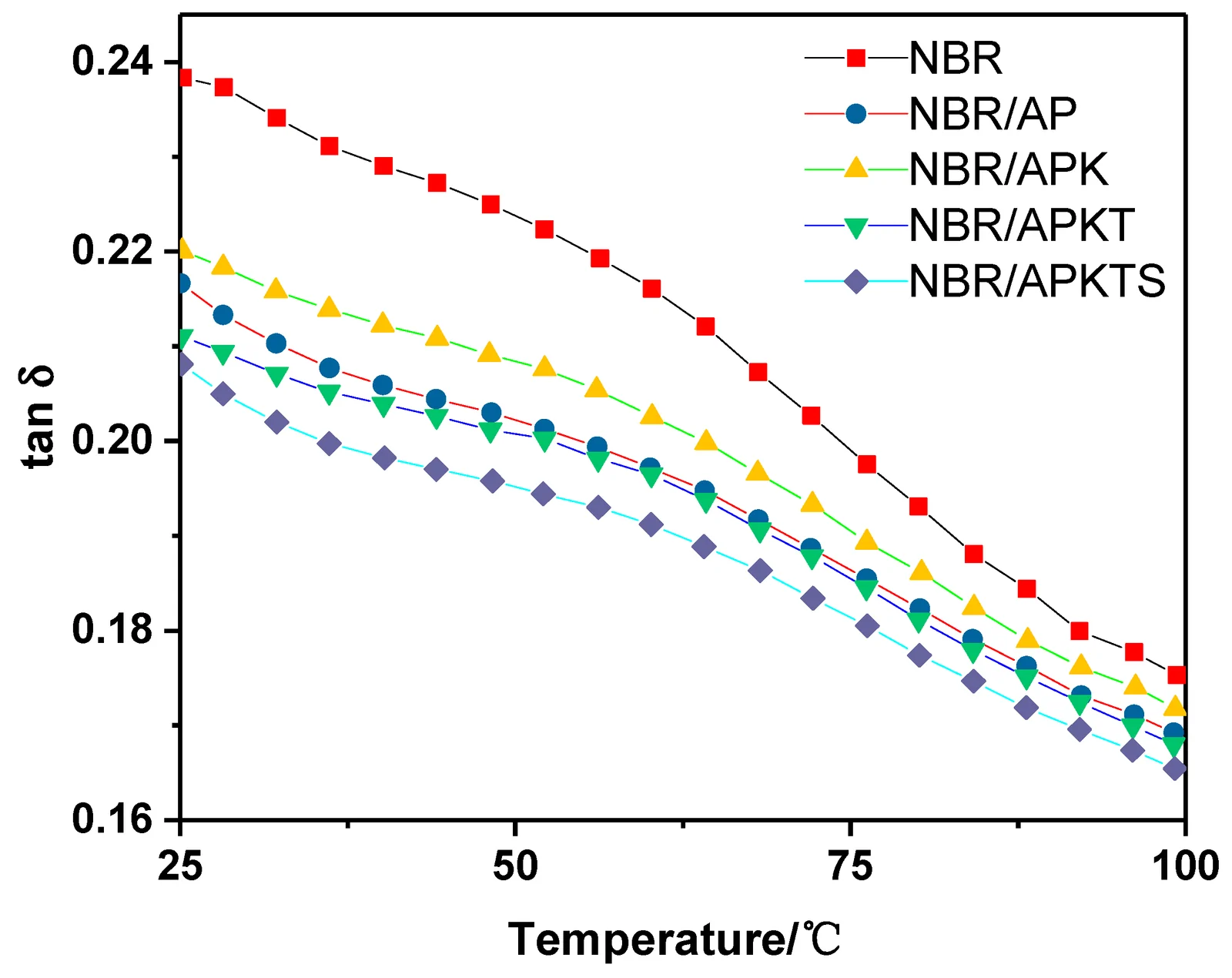
Temperature dependence of the tan δ for NBR reinforced with AP under different treatment processes.

**Table 1:** Formulations of the NBR compounds.

Ingredient (phr)	NBR	NBR/AP	NBR/APK	NBR/APKT	NBR/APKTS
Raw rubber (NBR), 3445C	104	100	100	100	100
Stearic acid	1	1	1	1	1
ZnO	5	5	5	5	5
Carbon black, N330	30	30	30	30	30
Carbon black, N550	30	30	30	30	30
Plasticizer, TOTM	8	8	8	8	8
AP (based on pure AP)	0	1 (AP)	1 (APK)	1 (APKT)	1 (APKTS)
Antioxidant, RD	1.5	1.5	1.5	1.5	1.5
Sulfur, IS-80	0.7	0.7	0.7	0.7	0.7
Accelerator, TMTD	0.5	0.5	0.5	0.5	0.5
Accelerator, DM	1.5	1.5	1.5	1.5	1.5

**Table 2:** Fitted C 1s peak areas and area ratios.

	O-C=O	C=O	C-N	C-Ph	O-C=O/C-Ph	C=O/C-Ph
AP a.u.	7203	8919	33,660	31,894	0.23	0.28
APK a.u.	12,241	6220	29,649	22,430	0.55	0.28
APKT a.u.	11,081	10727	28,695	30,365	0.36	0.35
APKTS a.u.	11,486	9738	31,854	23,530	0.49	0.41

**Table 3:** Fitted O 1s peak areas and area ratios.

	O-C	O=C	O-C/O=C
AP a.u.	8374	20,932	0.40
APK a.u.	11,649	18,196	0.64
APKT a.u.	10,728	19,066	0.56
APKTS a.u.	14,103	20,477	0.69

**Table 4:** The surface atomic percentages of the samples determined by XPS.

	C/at.%	N/at.%	O/at.%	S/at.%
AP	78.23	9.64	11.87	0.26
APK	74.58	12.46	12.68	0.27
APKT	75.01	12.57	12.09	0.33
APKTS	74.04	11.96	12.93	1.07

**Table 5:** Curing characteristics of aramid pulp-reinforced NBR subjected to different treatment processes.

	t_10_ m:s	t_90_ m:s	M_L_ dN·m	M_H_ dN·m	tan δ
NBR	0:42	2:03	0.93	14.28	0.0320
NBR/AP	0:50	1:46	1.00	14.07	0.0562
NBR/APK	0:49	1:43	1.01	14.17	0.0542
NBR/APKT	0:50	1:49	1.03	14.12	0.0553
NBR/APKTS	0:49	1:46	1.02	14.29	0.0572

**Table 6:** Mechanical properties of NBR reinforced with AP under different treatment processes (data are presented as median ± standard deviation, *n* = 3).

	NBR	NBR/AP	NBR/APK	NBR/APKT	NBR/APKTS
100% Modulus/MPa	3.6 ± 0.0	4.1 ± 0.1	4.1 ± 0.1	4.6 ± 0.1	5.4 ± 0.1
200% Modulus/MPa	10.0 ± 0.1	9.2 ± 0.1	8.9 ± 0.1	10.1 ± 0.1	11.9 ± 0.2
300% Modulus/MPa	17.1 ± 0.1	16.0 ± 0.2	15.5 ± 0.2	16.6 ± 0.2	19.9 ± 0.2
Tensile Strength/MPa	25.4 ± 0.1	23.0 ± 0.3	22.8 ± 0.4	23.1 ± 0.6	26.5 ± 0.3
Elongation at Break/%	445 ± 5	416 ± 12	418 ± 10	411 ± 9	394 ± 11

**Table 7:** Right-angle tear strength of NBR reinforced with AP under different treatment processes.

	NBR	NBR/AP	NBR/APK	NBR/APKT	NBR/APKTS
Tear Strength N/mm	55	58	57	58	59
Standard Deviation	2.2	2.5	3.9	2.6	2.3

**Table 8:** Crosslink densities of the samples determined by low-field nuclear magnetic resonance.

	NBR	NBR/AP	NBR/APK	NBR/APKT	NBR/APKTS
Crosslink density/mmol·cm^−3^	0.255	0.266	0.269	0.259	0.277
Standard Deviation	0.011	0.012	0.019	0.016	0.018

**Table 9:** Akron abrasion loss of AP-reinforced NBR subjected to different treatment processes.

	NBR	NBR/AP	NBR/APK	NBR/APKT	NBR/APKTS
Abrasion Loss (cm^3^)	0.93	0.85	0.88	0.86	0.82
Standard Deviation	0.007	0.012	0.009	0.025	0.011

**Table 10:** Compression heat build-up test results of AP-reinforced NBR under different treatment processes.

	NBR	NBR/AP	NBR/APK	NBR/APKT	NBR/APKTS
Temperature Increase °C	46.17	44.93	45.41	45.16	43.80
Standard Deviation	0.18	0.26	0.15	0.21	0.24

## Data Availability

The data that support the findings of this study are available from the corresponding author upon reasonable request.
